# Molecular subtypes based on DNA promoter methylation predict prognosis in lung adenocarcinoma patients

**DOI:** 10.18632/aging.104062

**Published:** 2020-11-24

**Authors:** Shanping Shi, Mingjun Xu, Yang Xi

**Affiliations:** 1 Diabetes Center, Zhejiang Provincial Key Laboratory of Pathophysiology, Institute of Biochemistry and Molecular Biology, School of Medicine, Ningbo University, Ningbo 315211, China

**Keywords:** lung adenocarcinoma, CpGs, DNA methylation, TCGA, the prognostic prediction model

## Abstract

Background: The heterogeneity of lung adenocarcinoma (LADC) makes the early diagnosis and treatment of the disease difficult. Gene silencing of DNA methylation is an important mechanism of tumorigenesis. A combination of methylation and clinical features can improve the classification of LADC heterogeneity.

Results: We investigated the prognostic significance of 335 specimen subgroups of Lung adenocarcinoma based on the DNA methylation level. The differences in DNA methylation levels were related to the TNM stage classification, age, gender, and prognostic values. Seven subtypes were determined using 774 CpG sites that significantly affected the survival rate based on the consensus clustering. Finally, we constructed a prognostic model that performed well and further verified it in our test group.

Conclusions: This study shows that classification based on DNA methylation might aid in demonstrating heterogeneity within formerly characterized LADC molecular subtypes, assisting in the development of efficient, personalized therapy.

Methods: Methylation data of lung adenocarcinoma were downloaded from the University of California Santa Cruz (UCSC) cancer browser, and the clinical patient information and RNA-seq archives were acquired from the Cancer Genome Atlas (TCGA). CpG sites were identified based on the significant correlation with the prognosis and used further to cluster the cases uniformly into several subtypes.

## INTRODUCTION

Lung cancer incidence rate and the associated mortality are among the highest in cancer. Global data shows that every year about 1.5 million deaths happen due to lung cancer, which is a mortality rate of above 25% [1]. Lung cancer is categorized into non-small cell (NSCLC) and small cell lung cancer (SCLC) based on their pathology, where NSCLC contributes to 85% of the cases [2]. LADC is becoming the predominant subtype of NSCLC, with the incidence rates increasing in recent years [3, 4]. Due to the resistance against radiation therapy, surgery remains the main treatment of LADC, but the five-year survival rate is low [5]. About one-third of the patients have a recurrence within five years of surgery, and the prognosis is not satisfactory [6]. LADC is clinicopathologically and molecularly heterogeneous, i.e., it responds differently to chemotherapy within the molecular subtypes leading to various prognostic values. Thus, it is of paramount importance to predict the outcome of a patient accurately [7, 8].

New evidence suggests that the associated effects of both genetics and epigenetics alternations have to be considered in tumorigenesis [9]. The oncogene mutation is no longer just an inherited or an epigenetic change. DNA methylation is a significant form of epigenetic alteration that is crucial for the expression of genes and often occurs on CpG islands, causing changes at the level of gene transcription [10–12]. Mounting evidence demonstrates that DNA methylation is the secondary “motive” for tumor occurrence following the genetic mutations, which proves that it is an important biomarker for early detection of tumors [13, 14]. Toyooka et al. [15] showed that DNA methylation is ubiquitous in all the stages of lung cancer initiation and progression with a negative regulatory effect on both oncogenic and tumor-suppressive gene expressions. Previous studies have shown that some gene methylation changes in LADC affect the gene expression and its prognosis [16, 17]. Thus, many researchers are now exploring methylation related biomarkers. Sandoval et al. [18] proposed a signature pattern with prognostic values based on five hypermethylated genes in the early stages of NSCLC. Also, Kuo et al. [19] developed a proof-of-concept signature pattern with prognostic potential based on eight methylated genes for survival outcome prognostication among Asian and Caucasian populations in the early stages of LADC.

We established a prognostic model to predict various DNA methylation markers through high-throughput omics analysis, which can advance the prognostic assessment and precision therapy.

## RESULTS

### Identification of overall survival -correlated prognostic methylation sites using the training dataset

TCGA DNA methylation profiling of LADC was exploited to cluster the LADC prognostic molecular subtypes. Firstly, the numbers for data pre-processing was optimized, which included absent value adaptation, removal of batch effect, sex chromosome, and single nucleotide polymorphisms, and the CpG sites in promoter regions extraction (Materials and Methods). For every CpG site obtained from the training set (generated from 335 tumor tissues), a univariate COX proportional risk regression model was established using the methylation status of the CpG sites and patient survival outcomes. The analysis resulted in 1302 CpG sites that were significantly correlated with the patients’ survival (p<0.05). Then, these CpG sites were entered into a multivariate COX proportional risk regression model combining the age, gender, TNM classification, and clinical stages as covariates to determine the independent prognostic features. Eventually, from both the regression models, 774 CpG sites were chosen and exploited as the conclusive classification characteristics (Supplementary Table 1).

### Identification of distinct DNA methylation prognosis subgroup by consensus clustering and inter-cluster prognosis analyses

**Figure 1:**
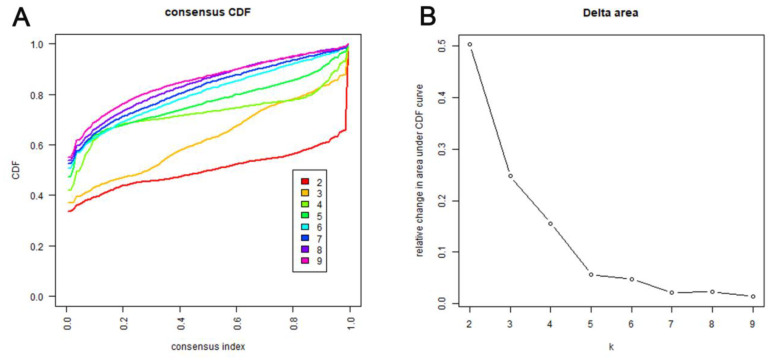
**Criteria for selecting number of categories.** (**A**) Consensus among clusters for each category number k. This graphic shows the cumulative distribution functions (CDF) of the consensus matrix for each k (indicated by colors), estimated by a histogram of 100 bins. This figure allows a user to determine at what number of clusters, k, the CDF each is an approximate maximum, thus consensus and cluster confidence is at a maximum at this k. (**B**) Delta area curves for consensus clustering indicating the relative change in area under the CDF curve for each category number k compared to k-1. The horizontal axis represents the category number k and the vertical axis represents the relative change in area under CDF curve. This plot allows users to determine the relative increase in consensus and determine k at which there is no appreciable increase.

**Figure 2:**
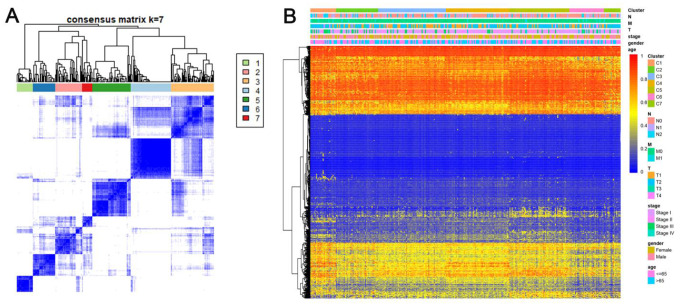
**Consensus matrix for DNA methylation classification with the corresponding heat map.** (**A**) Color-coded heatmap corresponding to the consensus matrix for k=7 obtained by applying consensus clustering. Color gradients represent consensus values from 0–1; white corresponds to 0 and dark blue to 1. To aid analysis, the cluster memberships are marked by colored rectangles between the dendrogram and heatmap according to a legend within the graphic. This enables a user to compare a clusters’ member count in the context of their consensus. (**B**) A heatmap corresponding to the dendrogram in (**A**) was generated using the heatmap function with DNA methylation classification, TNM stage, clinicopathological stage, age, and gender as the annotations.

Consensus clustering of 774 prospective prognostic methylation sites was performed to determine the distinguishable DNA methylation-based molecular subtypes of LADC for the prognosis. Several clusters were identified based on the following criteria: comparatively high consistency within the cluster with no apparent rise in area under the CDF curve. Based on the category numbers, we determined the cluster consensus average and coefficient of variation within the clusters. The area under the CDF curve becomes steady after five categories (Figure 1A, 1B). To advance the prognostic potential of LADC classifications, greater cluster numbers were selected whenever feasible. A consensus matrix was further exploited (mentioned in the Materials and Methods) to define the ideal cluster numbers. The consensus matrix displayed in Figure 2A indicates k = 7 consensus and a seven-block structure was identified. Heatmap, as per the dendrogram, is shown in Figure 2A with TNM category, stage, age, gender, and DNA methylation subtype, while annotations are displayed in Figure 2B.

**Figure 3:**
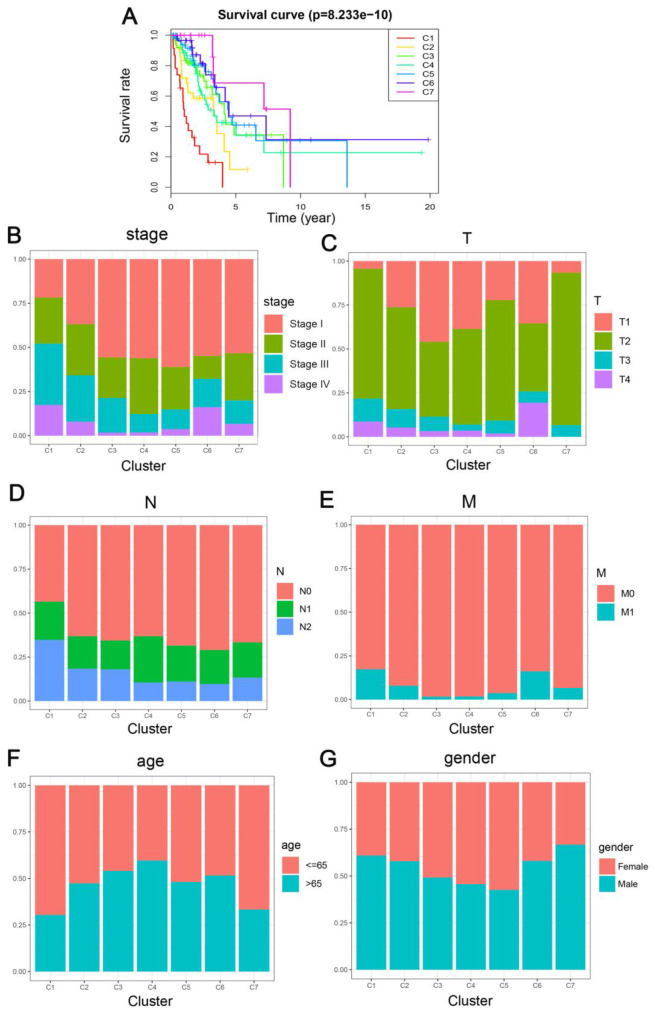
**Comparison of prognosis, TNM stage, age, and gender between the DNA methylation clusters.** (**A**) Survival curves for each DNA methylation subtype in the training set. The horizontal axis represents survival time (years), and the vertical axis represents the probability of survival. The log-rank test was used to assess the statistical significance of differences between subtypes. Stage score (**B**), topography score (**C**), lymphocyte infiltration (**D**), metastasis (**E**), age (**F**), and gender (**G**) distributions for each DNA methylation subtype in the training set. The horizontal axis represents the DNA methylation clusters.

Kaplan-Meier survival curve exhibited a considerable difference in the outcomes of the seven clusters (P<0.05). As revealed in Figure 3A, Clusters 6 and 7 showed the most promising prognosis, while, cluster 1 was the least promising. Next, we examined the intra-cluster fractions for the 7 clusters according to the stage (Figure 3B), TNM category (Figure 3C–3E), age (Figure 3F), and gender (Figure 3G), respectively. Predilections for correlations between features and certain clusters are listed below: Clusters 1 and 2 were correlated with the advanced stages; Clusters 3, 4, and 6 with lower T grade; while Cluster 1 was correlated with higher N grade and along with Cluster 6 it was related to higher M grade as well; Cluster 4 was associated with older ages; Cluster 5 was correlated with more number of females. This elaborated the rationale that cluster 1 showed the worst prognosis since it was more predisposed to disseminate and progress the malignancies while exhibiting a similar etiology as the DNA methylation aberrations. These data demonstrate that every single clinical feature corresponded to a different intra-cluster fraction.

### Identifying the features by DNA methylation clustering and screening the cluster-specific methylation sites

**Figure 4:**
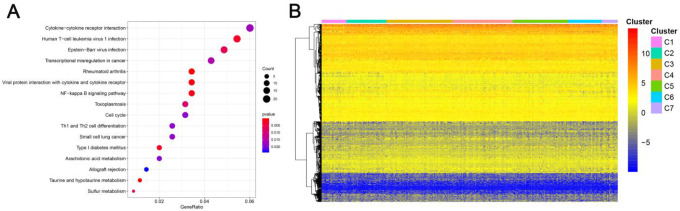
**Gene annotations of 774 methylated sites.** (**A**) KEGG function enrichment analysis of annotated genes. The graph's horizontal axis shows the gene radio and the vertical axis shows different gene functions. The dot size is proportional to gene count and p value is indicated by color. (**B**) Cluster analysis heat map for annotated genes associated with the CpG sites.

Genomic level annotations for the outlined 774 CpG sites were exploited to locate the associated 893 promoter related genes in total. Subsequently, we performed the functional enrichment analysis for these 893 genes and identified 16 dramatically enriched pathways (P<0.05), as exhibited in Figure 4A and Supplementary Table 2. The three most considerably enriched pathways included rheumatoid arthritis, Taurine and hypotaurine metabolism, and viral protein interaction with cytokine and cytokine receptors. Later, we examined the expression level of the methylated genes determined in the subgroups. The heatmap of gene expression is displayed in Figure 4B, and the raw data are listed in Supplementary Table 3. The patterns of gene expression diverged among different subtypes, implicating that DNA methylation levels represent corresponding gene expressions.

**Figure 5:**
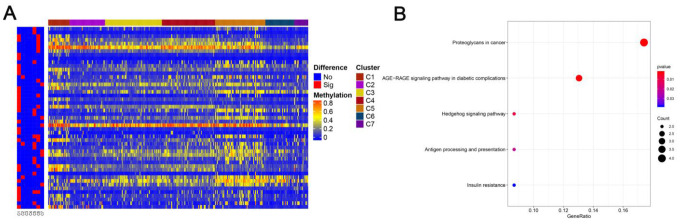
**Specific methylation CpG sites for each DNA methylation cluster.** (**A**) Specific CpG sites are shown for each DNA methylation prognosis subtype. Red bars represent specific CpG sites with significant differences. (**B**) KEGG pathway enrichment analysis of specific CpG sites.

**Figure 6:**
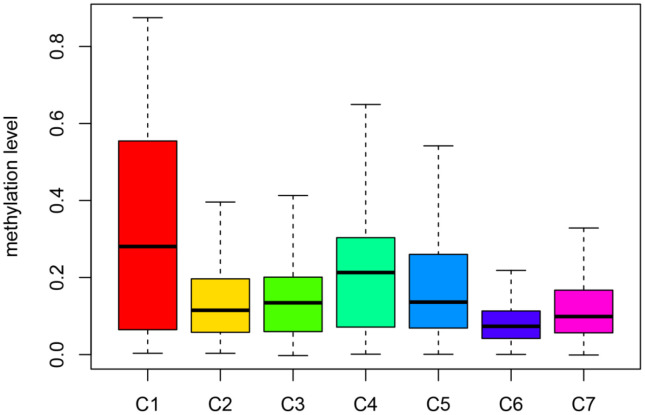
**Box plot of CpG methylation levels of the 7 Clusters.** Cluster 6 has the lowest CpG methylation level and Cluster 1 has the highest CpG methylation level.

Then, we filtered the cluster-specific methylation sites by referring them as features of corresponding clusters. Firstly, the differences within the 7 clusters were evaluated for each methylation site, as illustrated in Materials and Methods. The resultant, 61 cluster-specific methylation sites are listed in Supplementary Table 4 along with the heatmap in Figure 4A. Cluster 6 exhibited the best prognosis with 13 particular sites, all of which showed hypomethylation, and their methylation status was the lowest compared to all other clusters (Figure 6). Genomic annotations were applied to these 61 particular sites to define their match-up genes. ClusterProfiler analyses revealed the genes that were enriched in the five pathways, displayed in Figure 5B (Supplementary Table 5). These data revealed that each cluster possesses distinctive gene expression signature and pathway features.

### Establishment and evaluation of the LADC prognosis predicting platform

We chose Cluster 6 as a seed cluster since it was associated with a good prognosis and then established the Cox Proportional Hazard Model according to the methylation status profiling of the 18 specified sites integrated with the prognosis records using formulae described in Materials and Methods. Finally, five methylation sites (cg03476195, cg03699566, cg07572341, cg14896516, cg19224164) in hazard ratio model were identified. Subsequently, we developed a risk score equation: Risk score = 1.3247 × β value of cg03476195 + 2.3568 × β value of cg03699566+ –6.9075 × β value of cg07572341+ –6.9075 × β value of cg14896516+ 1.3834 × β value of cg192241646.

**Figure 7:**
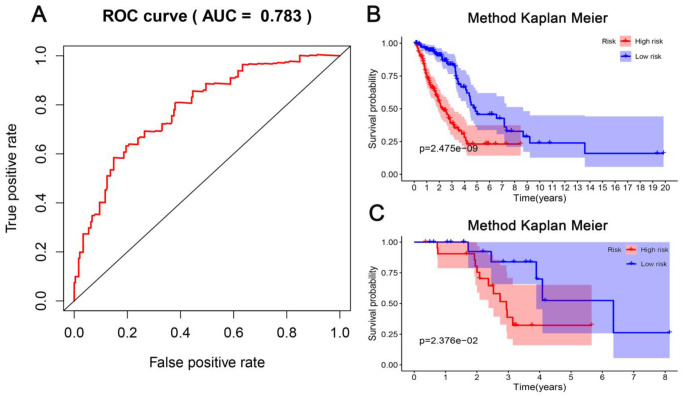
**Evaluation of the predictive performance of the model.** (**A**) Receiver operating characteristic (ROC) analysis of the sensitivity and specificity of the survival time by the five CpG sites in the training dataset. (**B**) The Kaplan-Meier analysis was used to visualize the survival probability for the low-risk versus high-risk group of patients based on the median risk value in the training dataset. Rows represent survival time (years), and columns represent survival rate. (**C**) Verification in the testing dataset with the Kaplan-Meier analysis.

**Figure 8:**
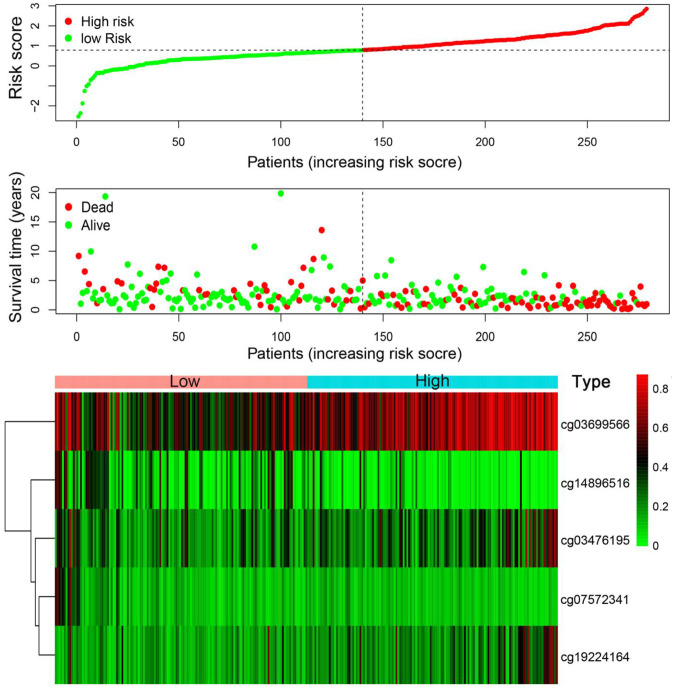
**Risk score analysis of the training set.** The five DNA methylation signature risk score distribution. Heat-map of the DNA methylation profiles. Rows represent CpG sites, and columns represent patients.

According to the risk scoring formula, we conducted the ROC analysis on the risk scores of each sample, as displayed in Figure 7A. The area under the curve (AUC) was 0.783, which indicated that this platform performs well in predicting prognostic outcomes. Next, we classified the patients into high- and low-risk subgroups with the median risk as a dividing line. Through Kaplan-Meier survival analysis, we found that the patients in high-risk subgroup showed drastically poorer outcomes compared to those in the low-risk group (Figure 7B), which was also verified in the test group, indicating the predictive reliability and durability of this platform. (Figure 7C). Additionally, we ranked the samples according to the risk scores to specify if the level of methylation changed regularly with the risk scores. Figure 8 shows a scatter plot of the risk score distribution and the patient status, where high risk is related to more deaths. The Heatmap showed a comparison of the methylation status of the five methylation sites between the high- and low-risk groups. The methylation levels of cg03699566, cg03476195, cg19224164 increased with the increasing risk, while the methylation levels in cg14896516 and cg07572341 increased with the decreasing risk.

## DISCUSSION

LADC is a common histological subtype, with high mortality and poor prognosis [20]. At present, the treatment of LADC is mainly surgery. However, nearly half of the patients show recurrence or die after the operation, resulting in a low 5-year survival rate [21]. To improve the management of LADC, it’s crucial to discover new prognostic markers that could facilitate outcome assessment, molecular subtyping, staging, prediction of relapse, and successful early care and medications. Epigenetically, gene silencing through DNA methylation was recognized as a significant mechanism during tumorigenesis [22]. Therefore, it is appropriate to presume that the DNA methylation status of some genes can be a useful biomarker to predict the tumor’s behavior.

Currently, emerging identifications of diverse methylations located at the gene promoter regions of certain genes are correlated with both the initiation and development of LADC. Also, alterations at the epigenetic level were seen arising much before the alterations at the genetic level, in LADC. At this moment, DNA methylation at certain sites was showing an underlying association with the initial pathogenesis of LADC. Lissa et al. [23] found that HOXA9 promoter methylation alone or along with the Blood Vessel Invasion (BVI) can act as a prognostic classifier. Some studies predicted the prognosis by combining the genes. Gao et al. found 118 differentially expressed methylation-regulated genes in both, LADC and the adjacent tissues and then integrated the expressions of genes for further exploitation as independent prognostic biomarkers or pharmaceutic targets for LADC [24]. However, the exact methylated sequences at the promoter regions remain unclear, and whether these gene methylations are clinically relevant to cancer classification, survival and outcome are still undetermined in a large cohort of LADC patients, which requires further validation. We endeavored to overcome these problems by establishing a classification platform that assembles multiple DNA methylation markers for prognostic assessment of curative efficiency while providing the therapy. This platform could assist in identifying novel biomarkers, therapeutic targets for personalized medicine, and molecular classification of subgroups in LADC. The model might also facilitate outcome prediction, early diagnosis, as well as the management of patients, who belong to distinct epigenetic subgroups of LADC. Further, as one of the most important epigenetic modifications, DNA methylation was found to play an important role in the occurrence and development of different cancers with an epigenetic heterogeneity between them. Yang Liu et al. screened out many reliable prognostic markers for different cancers (BRCA, COAD, ESCA, etc.) through the TCGA database, explaining the heterogeneity of cancer at the DNA methylation level [25], leading us to envision the potential applicability of our methods to other types of cancers as well.

Nevertheless, limitations exist in our study. Firstly, the prognostic prediction model requires validation in a larger sample cohort. Secondly, the construction of the prediction model needs to be improved by using a platform or other tools. We aim to look into the possibility of establishing a practical prognosis predicting system but as it’s still rudiment, further improvements are needed. Thirdly, this work was challenging to determine an optimal k in consensus. Collectively, we analyzed methylation data, clinical information, and the RNA-seq data of lung adenocarcinoma by various bioinformatic tools and discovered that certain methylation sites were significantly related to the prognosis. We also constructed the prognosis prediction model for LADC patients, which helps in identifying the novel markers and potential therapeutic targets for personalized medicine based on the molecular subgroups, eventually predicting the outcomes and assisting in early diagnosis, and also providing treatment to the patients who belong to the distinct epigenetic subgroups.

## MATERIALS AND METHODS

### Data collection and analysis

RNA-seq data from 594 cases of LADC were obtained from TCGA (https://cancergenome.nih.gov/, accessed 08 Mar 2020). The patient information, along with the follow-up records of 522 cases, is listed in Supplementary Table 6. Dataset of methylation was generated using Illumina Infinium HumanMethylation450 and 27 BeadChip arrays from 503 and 150 patients, respectively, through the UCSC website (http://genome.ucsc.edu/, accessed 08 Mar 2020. Only cases with the follow-up records of over 30-days were recruited in this study. The methylation status of every single site was exhibited as a β value, which starts at zero (non-methylation) and peaks at one (full methylation). Over 70% of the cases were with missing CpG sites and dismissed for the analyses. Cross-reactive genome CpG sites that were characterized in “Discovery of cross-reactive probes and polymorphic CpG in the Illumina Infinium HumanMethylation450 microarray” were eliminated as well. Additional CpG sites with no longer accessible raw data were imputed by k-nearest neighbors (KNN) imputation steps. ComBat algorithm in the sva R package [26] was applied to eliminate batch effects by assembling the entire DNA methylation array dataset integrating the batch and clinical information. Unsteady genomic regions, for example, the CpG sites located at sex chromosomes or single nucleotide polymorphisms (SNPs), were also excluded. Considering that methylation of the DNA promoter affects the gene expressions, we specifically assessed the CpG sites at the promoter regions. Promoters are well-characterized regions present 2 kilobases upstream and 0.5 kilobases downstream from the transcription starting site. We finally adopted the samples whose gene expression profiling was accessible. Overall, 479 samples, including 21,120 methylation sites, were selected for the analyses. All the cases were segregated into two cohorts: the training cohort (HumanMethylation 450 BeadChip data) and the testing cohort (HumanMethylation 27 BeadChip data).

### Determining the classification feature by COX proportional risk regression model

Preliminary data suggested that LADC molecular subtypes exhibited distinct prognostic outcomes among the analyzed cases; hence, CpG sites that considerably affected the survival outcomes were selected as classification signatures. Firstly, the univariate COX proportional risk regression model was established by integrating the methylation status of each CpG site, TNM category, age, stage, and survival information. Significant CpG sites selected through the univariate COX proportional risk regression model were then put into the multivariate COX proportional risk regression model utilizing the same covariates as in the univariate model, such as TNM category, age, and stage, etc. Eventually, the CpG sites that were significant in both the models were adopted as signature CpG sites.

### Correlation of molecular subgroups with prognosis determination by consensus clustering

Consensus clustering was implemented by the ConsensusClusterPlus package in R [27] to determine the LADC subtypes according to the most unstable CpG sites. The algorithm started through sub-sampling the proportions of items together with characteristics based on dataset matrix, where every sub-group was separated up to k groups by k-means. This step was repeated for multiple rounds set by the users, and multiplex clustering algorithm runs were utilized to determine the consensus values along with examining the constancy of recognized clusters. Pairwise consensus values specified as clustering fraction was established, out of which two items were pooled together, analyzed, and recorded in a consensus matrix for every k. Later, for every single k, an ultimate agglomerative hierarchical consensus clustering was finalized using the distance of 1-consensus values and pruned to k groups. This algorithm established the “consensus” clustering by examining the clustering outcome stability via applying a provided clustering approach to randomly selected data subunits. For every single iteration, 80% of the samples were selected, while exploiting the k-means algorithm with Euclidean squared distance metric. Similar outputs were compiled over 100 iterations. Following the implementation of ConsensusClusterPlus, we acquired the cluster consensus and item-consensus output. Graphical data incorporated the heatmaps of consensus matrices, which revealed the clustering data, consensus cumulative distribution function (CDF) plots, and delta area plots enabling us to define an approximate number of clusters. The Cluster numbers were defined based on the following standards: the ones with comparatively high consistency among the cluster, low coefficient of variation, and without an apparent increase in the area under the CDF curve. The coefficient of variation was computed based on the following equation: CV =(SD/MN)*100%, where SD stands for the standard deviation while MN is the sample number average. The number of categories was determined according to the area under the CDF curve but with no significant alteration. To generate further in-depth classifications of LADC, larger cohorts were preferably needed.

Heatmaps associated with the consensus clustering were produced by the pheatmap R package. Consensus values starting from zero (white) to one (dark blue) were represented by color-gradient mode, and a matrix was organized such that the samples correlating to a specific cluster were exhibited as adjacent. Here, the matrix correlated with a perfect consensus exhibiting a color-coded heatmap featured by blue blocks along the diagonals with a white background. The color-coded heatmap corresponding to the consensus matrix using consensus clustering is displayed in Figure 2A depicting the consensus for k = 7 by identifying the seven-block structure.

### Survival outcome and clinical feature analysis

Kaplan–Meier plots were generated to demonstrate overall survival within the LADC subtypes characterized by DNA methylation profiling. The Log-rank test was performed to examine the significant differences between the clusters. Survival outcomes were analyzed by survival packages in R. Correlations among clinical characteristics, and DNA methylation clusters were analyzed utilizing the chi-squared test. All analyses carried out were two-sided; P<0.05 was regarded as statistically significant for each analysis.

### Functional enrichment analyses and genomic annotation

We applied the cluster profile package in R [28] with KEGG for the gene enrichment analysis of Gene Ontology, Biological Pathways, and Regulatory motifs in DNA and Protein gene groups.

## Supplementary Material

Supplementary Table 1

Supplementary Table 2

Supplementary Table 3

Supplementary Table 4

Supplementary Table 5

Supplementary Table 6
