# Supplementary material for: Molecular subtypes based on DNA promoter methylation predict prognosis in lung adenocarcinoma patients
Source: Aging (Albany NY). 2020 Nov 24;12(23):23917–30. doi: 10.18632/aging.104062 (PMC7762488; doi:10.18632/aging.104062)
Supplement: Supplementary Table 2 [file aging-12-104062-s002.pdf]

SUPPLEMENTARY TABLE

Supplementary Table 2. Functional enrichment analysis and the identified 16 enriched pathways.

ID	Description	GeneRatio	BgRatio	pvalue	p.adjust	qvalue	geneID	Count
hsa05323	Rheumatoid arthritis	12/349	93/8017	0.000662	0.127121	0.120877	TGFB3/HLA-DOB/CXCL3/ATP6V1B1/CCL5/ATP6V1G3/HLA-DMA/MMP3/HLA-DRA/HLA-DPB1/ICAM1/CXCL1	12
hsa00430	Taurine and hypotaurine metabolism	4/349	11/8017	0.000913	0.127121	0.120877	GGT1/GGT6/GGT5/GAD2	4
hsa04061	Viral protein interaction with cytokine and cytokine receptor	12/349	100/8017	0.001271	0.127121	0.120877	CX3CR1/CSF1R/CXCL3/CCL5/TNFRSF10D/CXCL13/CXCL14/CX3CL1/CCL25/IL20/CXCL1/CCR8	12
hsa04064	NF-kappa B signaling pathway	12/349	104/8017	0.00179	0.134274	0.127678	LCK/PIDD1/CXCL3/BLNK/TRAF1/ATM/ICAM1/LY96/CXCL1/TAB1/TIRAP/CARD11	12
hsa04940	Type I diabetes mellitus	7/349	43/8017	0.002319	0.139159	0.132323	FASLG/HLA-DOB/PTPRN/HLA-DMA/GAD2/HLA-DRA/HLA-DPB1	7
hsa05166	Human T-cell leukemia virus 1 infection	19/349	219/8017	0.003147	0.157372	0.149642	PTTG2/LCK/MSX1/TGFB3/ANAPC2/HLA-DOB/HRAS/CDK2/CDC23/SLC25A31/E2F1/HLA-DMA/CD3G/HLA-DRA/HLA-DPB1/ATM/ICAM1/E2F2/VAC14	19
hsa05169	Epstein-Barr virus infection	17/349	201/8017	0.006557	0.281035	0.26723	HLA-DOB/PSMC5/CDK2/CD247/NFKBIB/USP7/RUNX3/E2F1/HLA-DMA/CD3G/BLNK/SKP2/HLA-DRA/HLA-DPB1/ICAM1/E2F2/TAB1	17
hsa00920	Sulfur metabolism	3/349	10/8017	0.00781	0.292863	0.278477	SUOX/PAPSS1/SELENBP1	3
hsa05145	Toxoplasmosis	11/349	112/8017	0.00933	0.310994	0.295717	TGFB3/HLA-DOB/LAMC1/NFKBIB/HLA-DMA/BIRC7/HLA-DRA/HLA-DPB1/LY96/HSPA6/TAB1	11
hsa05202	Transcriptional misregulation in cancer	15/349	186/8017	0.015514	0.380094	0.361422	SMAD1/RUNX2/HMGA2/CSF1R/PPARG/NUPR1/HHEX/PTK2/JUP/MITF/MMP3/TRAF1/ATM/WT1/LDB1	15
hsa04060	Cytokine-cytokine receptor interaction	21/349	294/8017	0.017276	0.380094	0.361422	FASLG/TGFB3/GDF5/CX3CR1/CSF1R/CXCL3/TNFSF18/IL1F10/CCL5/TNFSF15/TNFRSF10D/TNFSF12/CXCL17/CXCL13/CXCL14/CX3CL1/CCL25/IL20/IL32/CXCL1/CCR8	21
hsa04658	Th1 and Th2 cell differentiation	9/349	92/8017	0.018292	0.380094	0.361422	LCK/HLA-DOB/CD247/NFKBIB/RUNX3/HLA-DMA/CD3G/HLA-DRA/HLA-DPB1	9
hsa05222	Small cell lung cancer	9/349	92/8017	0.018292	0.380094	0.361422	LAMC1/CDK2/PTK2/E2F1/BIRC7/SKP2/TRAF1/FN1/E2F2	9
hsa04110	Cell cycle	11/349	124/8017	0.018992	0.380094	0.361422	PTTG2/TGFB3/ANAPC2/CDK2/CDC23/E2F1/CDC14A/SKP2/ATM/E2F2/MCM3	11

hsa00590	Arachidonic acid metabolism	7/349	63/8017	0.019005	0.380094	0.361422	CBR1/GGT1/ALOX15B/GGT5/PLA2G12B/PLA2G5/CYP2J2	7
hsa05330	Allograft rejection	5/349	38/8017	0.023414	0.424015	0.403186	FASLG/HLA-DOB/HLA-DMA/HLA-DRA/HLA-DPB1	5
