# Supplementary material for: Molecular subtypes based on DNA promoter methylation predict prognosis in lung adenocarcinoma patients
Source: Aging (Albany NY). 2020 Nov 24;12(23):23917–30. doi: 10.18632/aging.104062 (PMC7762488; doi:10.18632/aging.104062)
Supplement: Supplementary Table 5 [file aging-12-104062-s005.pdf]

SUPPLEMENTARY TABLE

Supplementary Table 5. Functional enrichment analysis and the enriched 5 pathways.

ID	Description	GeneRatio	BgRatio	pvalue	p.adjust	qvalue	geneID	Count
hsa05205	Proteoglycans in cancer	4/23/2020	204/8017	0.002465312	0.125199669	0.124467507	ANK2/IHH/SHH/COL1A2	4
hsa04933	AGE-RAGE signaling pathway in diabetic complications	3/23/2020	100/8017	0.002782215	0.125199669	0.124467507	SERPINE1/PRKCE/COL1A2	3
hsa04340	Hedgehog signaling pathway	2/23/2020	50/8017	0.008871195	0.266135836	0.264579486	IHH/SHH	2
hsa04612	Antigen processing and presentation	2/23/2020	78/8017	0.020716307	0.422194563	0.419725589	CTSS/HLA-DMA	2
hsa04931	Insulin resistance	2/23/2020	108/8017	0.037846858	0.422194563	0.419725589	PPARA/PRKCE	2
